# Discovery of barley miRNAs through deep sequencing of short reads

**DOI:** 10.1186/1471-2164-12-129

**Published:** 2011-02-25

**Authors:** Andreas W Schreiber, Bu-Jun Shi, Chun-Yuan Huang, Peter Langridge, Ute Baumann

**Affiliations:** 1 Australian Centre for Plant Functional Genomics, the University of Adelaide, South Australia 5064, Australia

## Abstract

**Background:**

MicroRNAs are important components of the regulatory network of biological systems and thousands have been discovered in both animals and plants. Systematic investigations performed in species with sequenced genomes such as Arabidopsis, rice, poplar and Brachypodium have provided insights into the evolutionary relationships of this class of small RNAs among plants. However, miRNAs from barley, one of the most important cereal crops, remain unknown.

**Results:**

We performed a large scale study of barley miRNAs through deep sequencing of small RNAs extracted from leaves of two barley cultivars. By using the presence of miRNA precursor sequences in related genomes as one of a number of supporting criteria, we identified up to 100 miRNAs in barley. Of these only 56 have orthologs in wheat, rice or Brachypodium that are known to be expressed, while up to 44 appear to be specifically expressed in barley.

**Conclusions:**

Our study, the first large scale investigation of small RNAs in barley, has identified up to 100 miRNAs. We demonstrate that reliable identification of miRNAs via deep sequencing in a species whose genome has not been sequenced requires a more careful analysis of sequencing errors than is commonly performed. We devised a read filtering procedure for dealing with errors. In addition, we found that the use of a large dataset of almost 35 million reads permits the use of read abundance distributions along putative precursor sequences as a practical tool for isolating miRNAs in a large background of reads originating from other non-coding and coding RNAs. This study therefore provides a generic approach for discovering novel miRNAs where no genome sequence is available.

## Background

Non-coding RNAs (ncRNAs) are known to be important regulators of post-transcriptional regulation of gene expression. They fall into a number of different classes, such as short-interfering RNAs (siRNAs), transacting siRNAs (tasiRNAs), Piwi-interacting RNAs (piRNAs), as well as microRNAs (miRNAs) (for reviews, see [1,2]). These classes of ncRNAs are distinguished by their biogenesis pathways and the classes of genomic loci from which they arise, as well as by their levels of conservation in related organisms [3].

miRNAs are single-stranded RNAs of approximately 21 nucleotides (nt) in length. In plants long primary microRNA transcripts (pri-miRNAs) are generated by RNA polymerase II and fold into imperfect stem loop structures. These pri-miRNAs are processed by the RNAse II enzyme DICER-LIKE 1 (DCL1) resulting in shorter folded RNAs, the precursor miRNAs (pre-miRNAs). A further cleavage step by DCL1 releases the miRNA/miRNA* duplex with a 2 nucleotide 3' overhang from the pre-miRNA, with the miRNA* being the near reverse complement of the miRNA derived from the hairpin [2]. The miRNA/miRNA* duplex is then exported into the cytoplasm where the miRNA* is degraded and the remaining mature 20-24 nt long miRNA is incorporated into an RNA-induced silencing complex (RISC) that has the ARGONAUTE1 (AGO1) protein at its core. While plant pre-miRNAs often contain only one mature miRNA, they can also harbor more than one mature miRNA. In this case they fold into several hairpins that are processed independently.

Two mechanisms for the regulation of target genes by miRNAs have been proposed: target mRNA cleavage and translational repression. Research to date suggests that the former mechanism appears to be more prevalent in plants [2]. However, more recent studies indicate that translational repression might be more prominent in plants than first thought [4,5]. In both cases, miRNAs direct RISC to recognise their target mRNAs based on perfect or nearly perfect antisense complementarity between the miRNAs and the mRNAs. The AGO1 protein mediates the target cleavage between nucleotides 10 and 11 of the miRNA.

While forward genetics led to the identification of the first miRNA genes, *lin-4 *and *lin-7 *in *Caenorhabditis  elegans *[6,7], the vast majority of plant miRNAs has been identified through small scale cloning [8,9] and computational methods [2]. More recently, next generation sequencing technologies (MPSS, 454 pyrosequencing and SBS) have been employed for high-throughput sequencing of short ncRNAs. The downstream analyses of the generated data have led to the discovery of many putative miRNAs in a variety of species [10-18].

Computational approaches to miRNA discovery in plants typically make use of a combination of RNA secondary structure analysis and sequence conservation of mature miRNAs among different species. For instance, Jones-Rhoades and Bartel [19] identified previously unreported miRNAs by computationally screening the Arabidopsis and rice genomes for inverted repeats that could be folded into secondary structures complying with criteria based on known pri-miRNAs. Putative miRNA sequences that were conserved between rice and Arabidopsis were further analysed using the program MIRCheck. Of 13 previously unreported conserved miRNAs between Arabidopsis and rice, 7 were experimentally validated. Similarly, Bonnet et al. [20] extracted intergenic regions from Arabidopsis and rice that contained conserved putative miRNA sequences as well as properly folded stem-loop structures in both species. Wang et al. [21] also extracted potential hairpins from intergenic regions of Arabidopsis. A filter based on GC content, loop-length and sequence similarity to putative miRNAs in the rice genome was then used to narrow down candidates.

Target-based algorithms that produce miRNA candidates using only a single genome were developed by Adai et al. [22] and Lindow and Krogh [23]. Adai et al. [22] selected potential candidates based on a scoring system for the miRNA-target similarity, the miRNA-miRNA* overlap and the minimum free energy (mfe) of the hairpin delimited by the miRNA-miRNA* pair. In addition to structural constraints, intergenic location, low copy number and stable miRNA/mRNA duplex formation, Lindow and Krogh's procedure [23] for selecting miRNA candidates also demands that candidates have more than one target. This is thought to be characteristic of most plant miRNAs [24]. More recently, Lindow et al. [25] modified the approach to include a support vector machine classification of miRNA precursors and identified a large number of putative miRNAs in Arabidopsis, Populus and rice, many of which are not conserved between species.

All approaches described above require the availability of fully sequenced genomes, which is not the case for the species of interest here. In cases where full genome sequences are not available, searching transcribed sequences such as expressed sequence tags (ESTs) has led to the identification of conserved miRNAs [26,27]. However, this data mining approach is very much restricted to abundant miRNAs, it requires a large EST data set and it can only result in identification of miRNAs previously identified in other species. Very recently, it has been employed by searching barley EST collections for homologs of known plant miRNAs obtained from miRBase [28]. In this way, Colaiacovo et al. could identify putative barley miRNAs belonging to 50 different miRNA families [28]. On the other hand, deep sequencing of small RNA libraries has enabled the discovery of non-conserved, conserved and low abundance miRNAs in a range of plant species [10,13-15,17,29-33]. Nevertheless, the analysis of this data is not without question, especially in the absence of the genome sequence.

Where the genome is available, a typical analysis pipeline of deep sequencing data includes steps that remove known RNA species such as messenger RNA (mRNA), ribosomal RNA (rRNA), transfer RNA (tRNA), small nuclear RNA (snRNA), small nucleolar RNA (snoRNA) and repeat associated small interfering RNA (rasiRNA) from the dataset, followed by mapping of the candidates to the genome. A range of subsequent criteria based on frequency in the genome, the analysis of the sequence structure surrounding the match for hairpin formation and sequence comparison to already identified miRNAs in other species are subsequently employed to identify miRNA candidates. In studies on plant species whose genome has not been completely sequenced, researchers have relied on available BAC sequences, genome survey sequence (GSS) databases and EST sequences for performing the candidate search [16,33].

We are interested in extending these studies to the grass species barley. Barley (*Hordeum vulgare L*.) is an important crop with an annual production of over 157 million tonnes across the world, on an area of over 56 million hectares. It is the fourth ranking cereal crop (http://faostat.fao.org, 2008). The barley genome is 5.4 Gb in size [34], almost twice the size of the human genome. Sequencing of this genome is currently being undertaken by the International Barley Sequencing Consortium [35], with an expected completion data of around 2012 (press release April 2008, http://prlog.org/10063090). Recently the gene content of barley chromosome 1H has been published [36].

We describe here a miRNA discovery project employing high-throughput sequencing of small RNAs from barley leaves. In addition to the usual filtering of reads to remove unwanted classes of RNA as well as precursor identification from available barley BAC and EST sequences, we also exploited sequence conservation among the grasses in order to make use of the more abundant wheat BAC as well as rice and Brachypodium genome sequence information. In this way we could gather evidence for the existence of many more miRNAs in barley than would otherwise be possible, given the limited sequence information for this species. We also made extensive use of characteristic read abundance distributions aligned to miRNA precursors as an additional tool for filtering out extraneous sources of short sequences, such as degradation products from mRNAs.

## Results

### Culling of reads with potential sequencing errors

**Figure 1:**
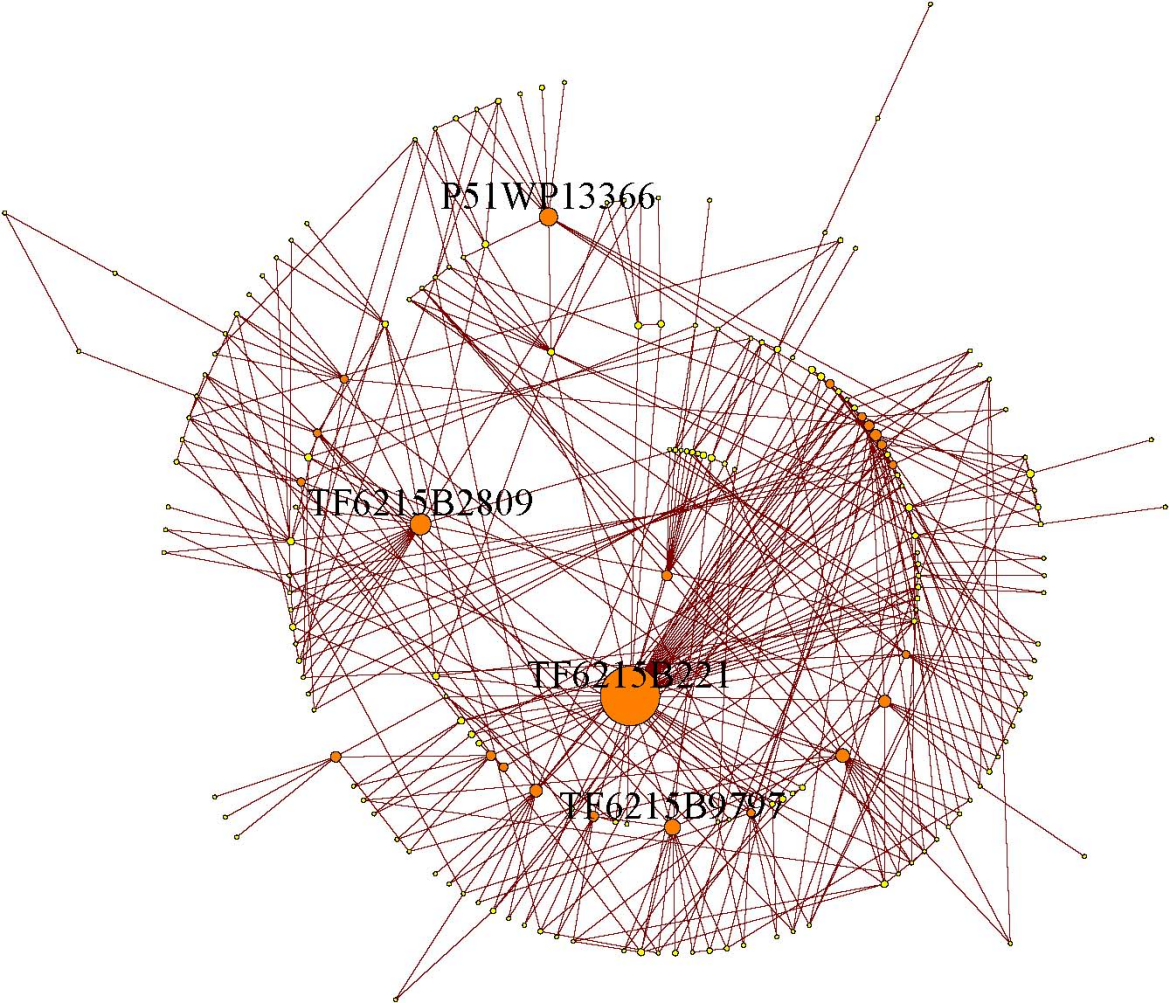
**Relation between sequences similar to TF6215B221**. Each vertex represents a unique sequence while each edge links two sequences differing by one base. The size of the vertex is related to the abundance of the corresponding sequence. This graph shows that TF6215B221, with an abundance of 5888, is accompanied by a large number of sequences differing by 1, 2 or more bases.

**Figure 2:**
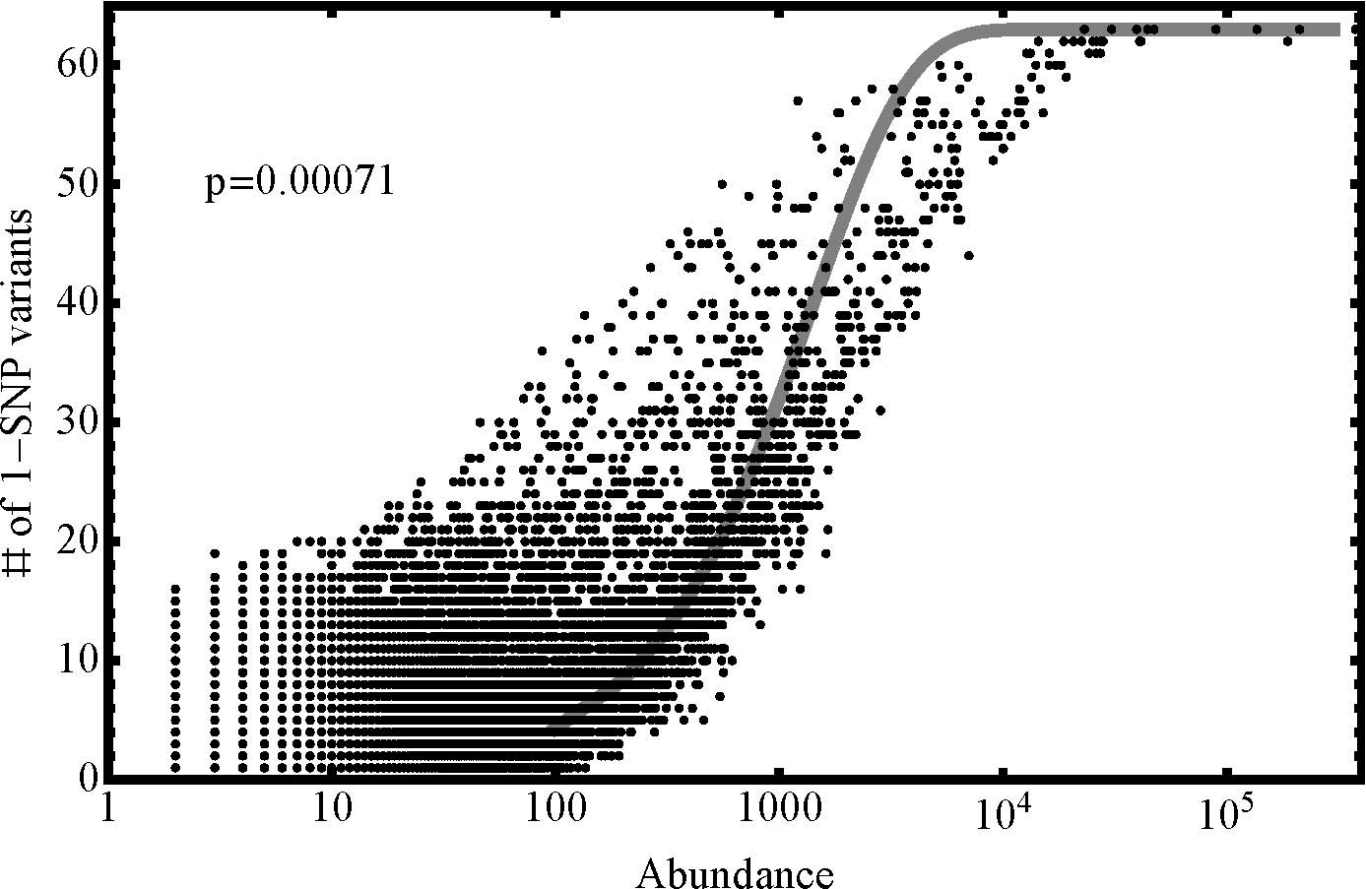
**The relationship between abundance of a sequence and the number of distinct 1-SNP variants thereof present in the dataset**. Any sequence with more than about 100 copies is accompanied by at least one variant, and the more abundant sequences are always accompanied by a large fraction of all possible variants. The curve represents a fit to the data as described in the Methods section.

Elimination of sequencing reads containing sequencing errors ('technical variants') is usually achieved by matching reads to a finished genome or, since this is not available for barley, through culling reads based on quality information from the sequencer. For transcript sequencing projects with extreme variation in read depth the latter is not possible because, while the probability of a single base substitution might be quite small, the abundance of the most abundant reads easily compensates for this. An inspection of the abundance distributions in our dataset bears out this expectation. For example, Figure 1 depicts a typical, moderately abundant, 'parent' sequence in our dataset (TF6215B221, abundance 5888). This sequence is accompanied by 54 less abundant 1-SNP variants (connected by an edge to the parent in the network depicted in Figure 1 with an average abundance of 12.2) as well as 92 2-SNP variants (average abundance 3.3). Even higher-SNP variants are apparent in Figure 1. Furthermore, as illustrated for sequences of 21 base-pairs in length in Figure 2 there is a strong correlation between read abundance and number of 1-SNP sequence variants. Indeed, we do not find a single sequence with abundance greater than 10000 that has fewer than 50 distinct 1-SNP variants. Furthermore, all the most abundant sequences depicted in this figure have the maximum number of 1-SNP sequence variants present, i.e. 63. The solid line in Figure 2 is the prediction (see Methods) one obtains if one assumes that all sequence variants are due to uncorrelated sequencing errors. As can be seen, the prediction fits the data rather well, which supports the conclusion that the bulk of the sequence variants in the dataset are of technical rather than biological origin. This quantitative agreement between theoretical expectation and the data provides clear evidence that sequencing error results in a proliferation of erroneous sequences of high abundance transcripts in next generation sequencing datasets. There is a clear danger, therefore, that technical variants of an abundant miRNA in a deep sequencing project can be misidentified as hitherto undiscovered family members and so identification and elimination of sequencing errors is vital to the analysis. As described in the Methods Section, we devised a method based on comparing relative read abundance of parent and variant sequences in order to remove sequencing errors from our dataset.

**Figure 3:**
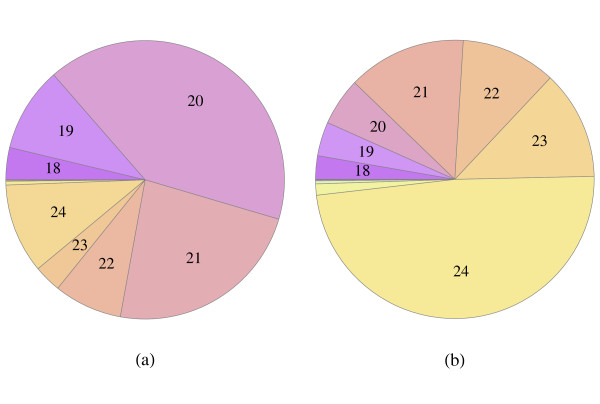
**Number of reads of fixed lengths**. Panel (a) depicts the redundant dataset while panel (b) shows the length distribution of unique sequences. 20, 21 and 24-mers are the most abundant sequences, while only a small number of reads are longer than 24 bases. 24 and 21-mers are the most frequent sequences in the non-redundant dataset. Note that those sequences with less than 18 bases have been removed from the dataset.

The length distribution of sequence reads with adapters trimmed off is shown in Figure 3. Reads of length 20, 21 and 24 are the most abundant, while only a small number of reads are longer than 24 bases. The most frequently observed read size is 20 (Figure 3a), with 71% of these being due to a single sequence matching an *Oryza sativa *tRNA-His gene. Almost half of the unique reads (Figure 3b) are 24-mers, the next most frequent group being 21-mers. Note, however, that apart from true miRNAs and the above-mentioned sequence variants, the dataset contains a multitude of sequences not related to miRNAs, such as other non-coding RNAs and mRNA degradation products. As described in the Methods section, we use standard techniques to eliminate these.

After taking into account the required secondary structure of putative miRNA precursors, as well as the distribution of reads along these precursors (see Methods), we ended up with 100 miRNA candidates that consisted of 56 with known homologs in other species and 44 putative novel miRNAs. Sequences for these miRNA candidates have been submitted to miRBase, and the primary sequence data can be accessed at the GenBank Short Read Archive under accession number SRA025074.1.

### miRNAs previously described in rice, wheat or Brachypodium

Previous studies have shown that some miRNAs are conserved across the plant kingdom, while others can only be found in a particular class such as the monocots. Some miRNAs may be specific to the grasses and others may even be newly evolved and species-specific. Availability of completed genome sequences for the dicot species Arabidopsis and poplar and the monocot rice allowed the identification of 20 miRNA families with members in all three species [2]. While sequenced genomes are still not available for most other monocot species, existing large EST and BAC sequence collections have been used for the identification of *Triticeae *miRNAs [28,37]. Recently, the rapid advances in high-throughput sequencing technologies have allowed the augmentation of these computational searches with valuable information on the actual expression levels of these miRNAs. For this reason, we have performed a comparison of the putative miRNAs contained in our dataset with those seen in similar experiments in rice, Brachypodium and wheat.

The specific datasets included in our comparison are shown in Table 1. In addition, we also included the results from a recent paper on Brachypodium miRNAs [38], which lists both experimentally confirmed miRNAs as well as computationally predicted miRNA candidates whose expression has not yet been confirmed. All mature miRNA sequences represented by more than 3 reads within any of these datasets [39] were compared and transitively clustered into miRNA families defined through a pairwise Levenshtein distance ≤ 3. Directly using sequences rather than names allocated by authors avoids complications due to inconsistent naming conventions in the literature, of which we found several.

**Table 1:** Data sources for evidence of expression of known miRNAs

Species	Reference	Tissues	Platform	Comments
Rice^a^	Sunkar et al. 2008 [31]	Seedlings	454, 18 K unique reads	control, droughted and salt stressed
Rice^a^	Zhu et al. 2008 [17]	7-day old shoots & roots, grain	454, 77 K unique reads; grain also with Solexa, 1.9 M unique reads	
Rice^a^	Xue et al. 2009 [32]	seeds	MPSS, 0.1 M unique reads	
Wheat^b^	Yao et al. 2007 [14]	leaves, root, spikes	454, 25 K unique reads	
Wheat	Wei et al. 2009 [33]	leaves, stem, root, young spikes	Solexa, 0.5 M unique reads	
Brachypodium	Wei et al. 2009 [33]	root, stem, leaves, spikes	Solexa, 0.6 M unique reads	
Barley	This study	leaf	Solexa, 3.6 M unique reads	

^a^Only rice miRNAs that are also listed in miRBase (V13.0, March 2009) were included in the analysis; miRBase names were used.

^b^Sequences from this reference as corrected in miRBase (c.f. miR164a and miR159b) were used; similarly, for miR501-515 and miR517-524 the revised names miR1117-1139 provided by miRBase were used.

Our dataset was searched for reads that could be grouped in with these families using the same criterion, but employing some additional refinements. In general, we consider only those reads that pass our filtering criteria (see Methods) and, for a given number of mismatches to a known miRNA sequence, only the most abundant read is retained. More importantly, our dataset contains a number of sequences that are simply length variants (differing by a single additional base at one end) or 'wobbles' (sequences of the same length but offset by one base). These variants can presumably arise simply because of inaccuracies of the action of DCL1 and hence are generally not regarded to each be individual miRNAs [39]. Typically we chose only the most abundant representative of such variants as a candidate. This approach is not followed universally (see, for example, Wei et al. [33]), which has led to differing estimates for the number of miRNAs in any particular miRNA family. The resulting barley counterparts for the miRNA families known to be expressed in rice, wheat or Brachypodium are listed in Additional file 1. Also shown in this table are the number of SNPs between our barley miRNA candidates and those in the three other grasses. In many, but not all, cases the mature miRNA sequence is conserved within all four species. Not listed in this table are those miRNA that are found in rice, wheat and/or Brachypodium, but not in barley. As discussed in more detail below, absence of a barley homolog of any particular miRNA should not necessarily be interpreted as absence of this miRNA from the barley transcriptome because our dataset only represents a single tissue, namely leaf.

### Families conserved between dicot and monocot species

Any miRNA that is known to have a hairpin precursor in *Arabidopsis *as well as at least one monocot we shall refer to as a miRNA "conserved between dicots and monocots". There are 21 such miRNA families that are known, of which 20 were described in Jones-Rhoades et al [2], while one, miR827, was described in Lacombe et al [40]. The datasets of Table 1 provide evidence on the number of distinct mature miRNAs that are transcribed from these loci in rice, wheat and Brachypodium. These results are compared with the number of mature barley miRNAs obtained in the present study in Table 2. As expected, there is evidence of transcription for most of these miRNAs in rice, wheat, Brachypodium and barley but there are also exceptions. Details for these are described below.

**Table 2:** The number of members of conserved miRNA families* in rice, Brachypodium, wheat and barley.

miR family	Rice	Rice transcribed	Brachypodium	Wheat	Barley
miR156, 157	3 (12)	3	10	4	2 (+2)
miR159, 319	6 (8)	5	12	11	2
miR160	3 (6)	3	5	2	2
miR162	2	2	0	0	0
miR164	4 (6)	4	3	3	3 (+1)
miR165, 166	6 (14)	6	8	7	4
miR167	2 (10)	2	6	5	4
miR168	2	1	3	3	6
miR169	9 (17)	9	9	10	5 (+1)
miR170, 171	5 (9)	4	5	8	2
miR172	3 (4)	3	6	6	0 (+2)
miR390, 391	1	1	1	1	1
miR393	2	2	3	3	1
miR394	1	1	1	0	0 (+1)
miR395	9 (23)	0	3	2	1
miR396	3 (5)	3	6	6	3 (+1)
miR397, 2029, 2508	2	2	4	3	2
miR398, 2025	2	1	2	2	0
miR399	7 (11)	2	5	2	4
miR408	1	1	2	1	0 (+1)
miR827	1	0	0	0	1

The entries refer to the number of mature miRNAs with unique sequences in the family, not the total number of miRNA loci in the family (for rice the number of known loci has been listed in brackets). Note that the misclassified novel miRNAs bdi-miR2508 and tae-miR2029 in Wei et al. [33] have been included as members of the miR397 family, while tae-miR2025 has been included as a member of the miR398 family. The numbers in brackets in the barley column refers to additional sequences present in the current study that were not included in our results because of sequence similarity to sequences in various repeat databases.

*By conserved miRNA families we mean those that are present in *Arabidopsis *and at least one monocot.

The miR162 family is represented by two members in rice, both of which were also found to be transcribed in Arabidopsis. However, no evidence of transcription for these two members was found in wheat [14,33], Brachypodium [33] or barley (Table 2). We have checked that in the case of barley, at least, this is not simply due to the filtering process: even in our unfiltered dataset, the most similar read to the two rice members of the miR162 family has four mismatches and even this is only represented by a single read. It is interesting to note that in rice [17,31,32] transcripts were found particularly in root tissue, but not in leaves or shoots. This may explain why miR162 is not seen in our barley data, which is extracted from leaf tissue alone. However, it does not explain why this miRNA is not seen in the published wheat and Brachypodium datasets, which contain transcripts from root tissue. On the other hand, in Arabidopsis miR162, which regulates production of DCL1 via a negative feedback loop [41], appears to be expressed in a range of tissues including leaves [41,42], while in rice it is not [17,31]. This indicates that tissue-specificity is not necessarily conserved across species.

The miR172, miR394 and miR408 families, which are transcribed in rice and are believed to regulate an APETALA2 transcription factor [43], a F-box protein [19] and a plantacyanin [44], respectively, are abundantly expressed in our unprocessed dataset. However, these sequences were subsequently eliminated by our filtering procedure because they are similar to known repetitive sequences. Specifically, the read similar to miR172 can be found (with ≤ 3 mismatches) in the Gypsy-class retrotransposon (Triticae Repeat Sequence Database entry TREP3208, *Triticum aestivum *sequence), the read that matches miR394 is similar to a number of Harbinger-class DNA transposon entries (e.g. TREP3044, *Oryza sativa *sequence) and the read that matches miR408 is similar to the Gypsy-class retrotransposon TREP2268 (*Triticum durum *sequence). While it might be appropriate to consider repetitive sequences as bona-fide miRNAs if other compelling evidence for their existence is available [39], we believe that in the absence of a genome sequence for barley it is better to err on the side of caution. While filtering of repetitive sequences had also been carried out for the Brachypodium and wheat datasets considered here [33], a less stringent filtering criterion was used. Hence, wheat and Brachypodium, but not barley, miRNA candidates for members of the miR172, miR394 and miR408 families are listed in Table 2.

The miR398 family, which is present in Arabidposis (3 members), rice (2 members), Brachypodium and wheat, is not present in our barley dataset. The datasets listed in Table 1 only provide evidence for the transcription of one of these members in rice [17,31,32], while two members are transcribed in both Brachypodium and wheat [14,33]. It is worth mentioning here that a similar sequence (with three-nt variation) to miR398 exists in wheat [33], but whether this is a member of the miR398 family requires experimental confirmation. In Arabidopsis miR398 targets Cu/Zn-superoxide dismutases CSD1 and CSD2 and is down-regulated under stress, particularly Cu-stress, conditions [45,46]. In this species, it has been shown to be highly expressed in cauline leaves and stem, lowly expressed in rosette leaves and is hardly expressed in floral tissues [45]. However, in another study it was found to be particularly highly expressed in rosette leaves [19]. In rice, a comparative study of expression in sprout, young panicle, young seeds, 3-week-old seedlings, un-differentiated and differentiated callus found this miRNA to be exclusively expressed in callus [47], whereas in Medicago it appears to be present in roots, stem, leaves and flowers [48]. In Brachypodium, no differential expression between vegetative and reproductive tissues has been reported [33]. In wheat it has not been detected in leaves, root and spikes [14]. In short, a consensus for the tissue dependence of the expression of miR398 within grass species has not yet been reached.

Another noteworthy feature of the data collated in Table 2 is that while miR395 is expressed in the wheat, Brachypodium and barley datasets, it does not appear in the three rice datasets reviewed in our study. This is the case even though the presence of the appropriate precursor to miR395 in the rice genome was used as evidence that it is among the 20 miRNA families conserved across monocots and dicots [2]. Indeed, the evolution and genomic organization of the rather large miR395 family have been investigated in detail in several rice species [49]. miR395 targets ATP sulfurylase and it is known that in Arabidopsis, at least, it is difficult to detect if sulfate levels are not limiting [19]. Therefore, we infer that the fact that expression of miR395 is detected in Brachypodium, wheat and barley but not rice may be because of low expression levels and comparatively small datasets for rice (see Table 1).

Finally, there is good evidence for the expression of a member of the miR827 family in the barley dataset. This miRNA targets proteins with a SPX motif associated with phosphate uptake. miR827 is a family that was not included in the original list of dicot/monocot conserved miRNAs in Jones-Rhoades et al. [2]. It is not expressed to any significant extent in any of the rice, wheat and Brachypodium datasets considered here but has been detected in rice in a separate study [40].

The relative abundance of the conserved miRNAs in the barley dataset was compared with that in other species. miR168 was found to be the most abundant miRNA, accounting for about 6.7% of the total sequence reads (Additional file 1). This is in agreement with the data from rice [31,50,51] and Brachypodium [52], but not Arabidopsis [51]. In Arabidopsis miR172 is the most abundant miRNA [53], while in barley miR172 is 30 times less abundant than miR168 (Additional file 1). Whether these two miRNAs are responsible for the difference between monocots and dicots is worth further investigation. It is known that miR168 regulates AGO1, which is involved in miRNA biogenesis. The extraordinarily high level of miR168 in monocots suggests strict regulation of the *AGO1 *mRNA [51]. miR172 promotes flowering [43,54,55], but its targets are still poorly characterized. Recent studies showed that miR172 acts downstream of miR156 and is regulated by miR156 [56]. Interestingly, miR156 is the second most abundant miRNA in the barley dataset, accounting for about 3.7% of the total reads (Additional file 1). This miRNA was also found to be the second most abundant miRNA in Brachypodium [52], but not in rice [51] and Arabidopsis [53]. These results, combined with the fact that Brachypodium is closer to barley than to rice [57,58], lead us to speculate that miR156 may have different or additional roles in barley and Brachypodium relative to rice.

### Families not conserved between dicot and monocot species

In addition to miRNA families conserved between the dicots and monocots, Jones-Rhoades et al. [2] noted that miR437, miR444 and miR445 have members in rice and maize, but not in Arabidopsis. This group of putatively 'monocot-specific' or possibly 'grass-specific' miRNA families is extended here by searching the rice, wheat, Brachypodium and barley datasets for any families that are expressed in at least two of these species but that have no known Arabidopsis homolog. For this, the Arabidopsis miRNA sequences used were taken from miRBase. Results are shown in Table 3 and, again, we indicate additional barley sequences that would be included in our dataset if we did not impose a stringent filtering of likely repetitive sequences.

**Table 3:** The number of members of monocot-specific miRNA families in rice, Brachypodium, wheat and barley.

miR family	Rice	Rice transcribed	Brachypodium	Wheat	Barley
miR437	1	1	1^a^		
miR444, 2024	6 (11)	4	5	4	1 (+4)
miR445	1 (9)	1			
miR528	1	1	1	1	1
miR910			1	1	
miR1135			1	1	0 (+1)
miR1318, 1432	2	2		1	2
miR2003				1	1
miR2009				3	5
miR2011				1	1
miR2032				1	1
miR2509			1		1

Included are those families classed as monocot-specific in [2] as well as those with evidence for expression in at least two grass species. The notation is the same as that for Table 2.

*^a ^*The evidence for miR437 comes from Unver et al. [38], who did not confirm expression for this miRNA.

miR437 is believed to target a glutamate receptor and shows moderate expression in rice leaves [44]. No expression is observed in leaf tissue of any of the other species considered here, nor was it seen in pooled maize tissues (including seedlings; see [44]). However, the conserved precursor sequence is known to be present in maize, sugarcane, sorghum [44] and Brachypodium [38]. In other words, the evidence for expression of miR437 in grass species beyond rice is presently unclear: the presence of a conserved sequence would indicate that it is functional. However, expression has not been confirmed.

On the other hand, there is clear evidence of expression of members of the miR444 family in all species considered here. Two sequences labelled as miR2024b and miR2024a [33] are actually identical to sequences labelled miR444b and miR444c in the same work, respectively, so we include them as part of the miR444 family. In our barley dataset, 4 members of this family are expressed, but three of these are sufficiently similar to retrotransposon and DNA transposon sequences contained in the TREP database for them to fail our filtering procedure for repetitive sequences. This is consistent with the findings in Sunkar et al. [31], who found that this MADS-box transcription factor-targeting miRNA family is present at multiple loci in the rice genome.

It is not surprising that we see no evidence for the expression of miR445 in our dataset, as it has previously been found to be strongly expressed in rice stems, but hardly detectable in rice leaves and inflorescence [44]. Similarly, miR910, which is an ancient miRNA identified in the unicellular *Chlamydomonas reinhardtii *[59], is not observed in our barley dataset, but has been observed both in Brachypodium and wheat [33]. We suggest that the observation by Wei et al. is likely spurious as only a single read supports its presence in Brachypodium and wheat [33]. In addition, one might consider it somewhat surprising to find a miRNA with identical sequence to the evolutionary distant *Chlamydomonas reinhardtii *in both Brachypodium and wheat, but not rice.

miR528, on the other hand, is detectable in all of the above species, while miR1318/1432 is seen in three out of the four species. Both miRNA families are therefore candidates for miRNAs conserved in at least a subset of monocot species. As far as we are aware, targets of miR528 have not been identified, while miR1432 is believed to target Ca^2+ ^binding EF-hand proteins [31].

miRNAs 2003, 2009, 2011 and 2032, expressed in the barley dataset, were all found to be expressed in wheat [33]. Reported predicted targets in wheat are: miR2003-large subunit of RNA polymerase II; miR2009-resistance proteins Yr10, Mla1 and Lr10; miR2011-putative MAPK and carbohydrate transporter; miR2032-FTL2. Surprisingly, analogous to the findings of Wei et al. [33], there is essentially no overlap between the monocot-specific barley miRNAs and the novel wheat miRNAs reported in Yao et al. [14].

Finally, we observe a relatively abundant sequence similar to bdi-miR2509 [33]. However, we believe that this is probably the miRNA* partner of miR166.

A number of miRNA-like reads were filtered out because they are too similar to known repetitive sequences for them to be identified as miRNAs. However, these sequences have been classified as miRNAs in other works. For this reason, we show in Table 4 those 'monocot-specific' candidate miRNAs that are eliminated by our filtering (see also Additional file 1) but have been included elsewhere. It is interesting to note that this difference in assessment between our work and the literature appears to be largely confined to the wheat data described in [33]. Table 5 on the other hand, lists those 'monocot-specific' miRNAs where expression in Brachypodium has not been confirmed [38]. Essentially none of these are present in our dataset.

**Table 4:** The number of members of monocot-specific miRNA families where barley reads were filtered out as repetitive sequences.

miR family	Rice	Rice transcribed	Brachypodium	Wheat	Barley
miR516				1	0 (+1)
miR530	2	1			0 (+1)
miR531	2	2			0 (+1)
miR1126				1	0 (+1)
miR1137				1	0 (+1)
miR1436	1	1			0 (+3)
miR2002				1	0 (+1)
miR2004				1	0 (+1)
miR2005				1	0 (+2)
miR2006				1	0 (+1)
miR2007				1	0 (+1)
miR2008				1	0 (+2)
miR2012				1	0 (+2)
miR2016				1	0 (+1)
miR2018				1	0 (+2)
miR2020				1	0 (+2)
miR2033				2	0 (+2)
miR2502			1		0 (+1)

Notation the same as that for Table 3.

**Table 5:** Additional members of monocot-specific miRNA families with unconfirmed expression in Brachypodium.

miR family	Rice	Rice transcribed	Brachypodium	Wheat	Barley
miR437	1	1	1		
miR1122			1	1	
miR1128, 1133			2	2	
miR1132			1	1	
miR1134			1	1	
miR1139			1	1	
miR1850	1	1	1		
miR1135			1	1	0 (+1)

Notation the same as that for Table 3.

### miRNAs expressed in barley, but not in rice, Brachypodium or wheat

A number of miRNA candidates in the barley dataset have sequences that appear unrelated to any rice, Brachypodium or wheat miRNAs that are known to be expressed. We refer to these, rather loosely, as 'novel' miRNAs. This interpretation needs to be qualified because, as described in the Background and Methods sections, we base our search strategy on a number of criteria, including the presence of a valid pre-miRNA sequence in available rice, wheat, Brachypodium or barley sequence databases. Hence, in most cases our miRNA candidates are associated with valid hairpin structures in another species. We nevertheless classify them as 'novel' because a) there is no known expression in other species and b) they have not previously been computationally predicted to be miRNAs in other grass species.

**Figure 4:**
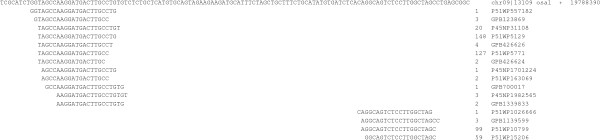
**The alignment of reads to the miR169j precursor region in rice is shown**. Note that the known miRNA and miRNA* (P51WP5129 and P51WP15206, respectively) are distinctive in being among the most abundant reads, while not a single read in the dataset aligns to the region excised by DCL1. Only perfect matches are shown, but reads with up to three mismatches were allowed and merely add to the two expressed regions.

These novel miRNA candidates are listed in Table 6. We associate various confidence levels with these candidates, depending on the characteristics of the read distribution associated with the candidate (see Methods). Clear, localized read distributions for miRNAs and associated miRNA*s analogous to those observed for well-known miRNAs described previously in rice (see Figure 4), are given the highest weight (***), followed by those where the localized read distributions are only observed for the miRNAs (**), but not the miRNA*s. The lowest confidence (*) is given to those candidates for which the read distributions, while still localized, are atypical. Note that all candidates have passed through our filtering procedure described earlier. Altogether, there are 44 candidates with abundances ranging from 16 to over 19000. The candidates GPA5819 and GPB2903 appear to belong to a single miRNA family, as do GPB2977 and GPB5902. While there is no evidence from our data that any of the other 40 candidates belong to multi-member miRNA families, it must be kept in mind that we have only probed one tissue.

**Table 6:** Candidates for novel barley miRNAs not previously described in rice, Brachypodium or wheat.

Read name	Proposed miRBase name	Read sequence	Read Length	Abundance	putative miRNA* name	putative miRNA* sequence	putative miRNA* Length	putative miRNA* abundance	Confidence
GPB235		AUAAAACCUUCAGCUAUCCAUC	22	4601	GPB7393	UUUGAUAUGUAAGUGGAUAGUU	22	431	***
GPB125	miR5048	UAUUUGCAGGUUUUAGGUCUAA	22	12068	GPB971	AGACCUAGACAUGCAAGUAUA	21	1201	***
GPB1131	miR5049	UCCUAAAUACUUGUUGUUGGG	21	803	GPB31055	AACAACAAGUAUUAUGGUACA	21	16	***
P51WP1692	miR408b	CAGGGAUGGAGCAGAGCAAGG	21	225	P51WP9508	UGCACUGCCUCUUCCCUGGC	20	48	***
GPB8582	miR5050	UUGAGGUCGUUCAACCAGCAA	21	78	P45NP15695	CAGCACUAGCAAGUUGGUCGACCU	24	34	***
GPB86	miR5071	UCAAGCAUCAUAUCAUGGACC	21	19176					**
GPA5819	miR5072	CGUUCCCCAGCGGAGUCGCCA	21	147					**
GPB2903	miR5054	UCCCCACGGACGGCGCCA	18	352					**
GPA3884	miR5055	UCUCGCUACUGAGCUCGGCAU	21	157					**
P45NP9164	miR5056	AGGAAGAACCGGUAAUAAGCA	21	72					**
P45NP15764	miR5073	GUUUGGUGAAUCGGAAACAAUUU	23	28					**
GPB4154	miR5057	AAAUUUCAGAUCAUUUGGACA	21	178					**
TF6215B7984	miR5058	AAUAGUUGAGGGAUGGAAAACA	22	68					**
TF6215B74145	miR5086	ACAUUGGUGGAAGGCGUGGUA	21	20					**
TF6215B6553	miR5074	GAAGGCCACCGUCGGCACCGC	21	142					**
P51WP57134	miR5059	CGUGCCUGGGCAGCACCACCA	21	16					**
P51WP4847	miR5075	UUCUCCGUCGACGCCAUCCGC	21	205					**
P51WP10727	miR5060	CGGCAAGCUAGAGACCGCCAC	21	68					**
P45NP51144	miR5061	UCUGUUCUGUUCUGAUCGGUA	21	24					**
P45NP40836	miR5051	UUUGGCACCUUGAAACUGGGA	21	21					**
P45NP31207	miR5076	UAAAUGGGAGCAGAGCAGGUUU	22	16					**
P45NP21816		UGAGCUACAAAAGGAUUCGUU	21	25					**
P45NP16270	miR1878	AUUUGUAGUGUUCGGAUUGAGUUU	24	33					**
GPB49459	miR5084	AUACAGUACUGCAGAGGAUCCUAA	24	16					**
GPB41819	miR5085	AAGGACAUUUUUUGUGGCAUG	21	47					**
GPB2977	miR5062a	UGAACCUUGGGGAAAAGCCGCAU	23	99					**
GPB5902	miR5062b	UGAACCUUAGGGAACAGCCGCAU	23	57					**
GPB16764	miR5063	UCCACUGGAAGAGGCUUUUGCU	22	54					**
P51WP18432	miR5052	ACCGGCUGGACGGUAGGCAUA	21	36					*
GPB1614	miR5064	UGAAUUUGUCCAUAGCAUCAG	21	513					*
GPB25359	miR5065	UAGGCAAUUCACAUAUACACU	21	27					*
GPB17570	miR5066	AAGUGUAUAUGUGGAUUGCCU	21	73					*
GPB26526	miR5053	CGCAGCUGUAGUCGCCGGCGU	21	22					*
GPB320	miR5077	GUUUCACGUCGGGUUCACCA	20	5056					*
P45NP187203	miR5078	GGUCGUUCGACCGCGGCAUUU	21	25					*
GPB373	miR5067	UGAGCGACAAGUAAUAUGGAU	21	4043					*
P45NP20958	miR5068	AUCAGGUAGAUCGGGUAUGGGUAU	24	40					*
P45NP15484	miR5079	UUUGGAUUUGUUAUUUUGGUAU	22	68					*
GPB9781	miR5080	AAAAAGAUCAUACCGUGAGAG	21	88					*
GPB37150	miR5069	UAGGUGAUUGAUUUGACUAAC	21	17					*
GPA47901	miR5081	UAAUUUGUAGCAAAUUGGUGU	21	16					*
GPA42571	miR5082	CGCGAUGAUGGCCGCGCGGGCUCA	24	17					*
GPA18850	miR5083	AGACUACAAUUAUCUGAUCA	20	28					*
GPA14470	miR5070	AACUAAGUAUGGUCGGAGGGU	21	49					*

As discussed, a necessary requirement for any read to be included in our list of miRNA candidates was the presence of a valid hairpin precursor in various barley, wheat, rice or Brachypodium sequence collections (see Methods). In addition, we have searched the known barley chloroplastic genome (gi|118201020) for sequences identical to our candidates. While we did not find matches for any of our miRNA candidates already known in other species, five of the novel miRNA candidates (P45NP9164, P45NP15764, P45NP31207, P45NP21816 and P45NP15484) could be found in this genome. As expected, corresponding hairpin precursors were not found in the chloroplast genome, so we see no evidence of miRNAs actually originating from the chloroplast genome. Given the fact that these five candidates do have corresponding hairpin precursors in the (nuclear) genome of other species we treat them as possible miRNA candidates. However, we caution that their additional presence in the barley chloroplast genome could indicate possible alternate mechanisms of biogenesis. In either case, it is interesting to note that all five of these candidates are predominantly expressed in the cultivar Pallas, but not in Golden Promise. Furthermore, they all appear to be more abundant under phosphorus-deficient conditions.

The novel miRNA candidates show a clear preference for a uracil at the 5' end because 20 of 44 candidates (i.e. 45%) start with this nucleotide. The dominance of uracil in this position is in agreement with previous findings in rice and Arabidopsis [27]. However, in the study of Zhang et al [52] the dominance was considerably higher (54%). Current figures (miRBase V13) for the frequency of U at the first position are: Arabidopsis 76% and rice 58%, while the second most common nucleotide is A, with Arabidopsis 12% and rice 11%. Similarly, our novel miRNA candidates have A as the second most common nucleotide at the 5' end, with a frequency of 36%.

Since the precursor sequences were taken from a number of different species, at the same time allowing for mismatches, there are often quite a few hairpin candidates for homologs of the barley precursor. These precursor homologs are listed in Additional file 2. The secondary hairpin structures of precursor exemplars are shown in Additional file 3.

Putative targets for these novel miRNA candidates were predicted using psRNATarget, http://bioinfo3.noble.org/psRNATarget[60,61], using the DFCI barley gene indices (http://compbio.dfci.harvard.edu/tgi/, Release 10.0) as a reference set. As is common for barley, the function of quite a number of the target genes is currently not known. A summary of those targets for which annotation of reasonable quality is available is shown in Table 7 with the full set listed in Additional file 4.

**Table 7:** Candidate targets for barley miRNAs not previously described in rice, Brachypodium or wheat.

Read name	Putative function (summary)
GPB235	Resistance gene? Nucleotide-binding site containing gene?
GPB125	Resistance gene? Serine/threonine kinase?
GPB1131	Triglyceride lipase-like? Resistance-related receptor?
P51WP1692	NADH-ubiquinone oxidoreductase-related-like? Fibroin? Protein kinase?
GPB8582	ETS-like protein?
GPB86	Nucleotide binding site containing resistance gene
GPA5819	RNA-binding glycine-rich protein
GPA3884	LysM receptor-like kinase
P45NP9164	DNA-directed RNA polymerase
GPB4154	aminotransferase?
TF6215B7984	Subtilisin-chymotrypsin inhibitor? Betaine aldehyde dehydrogenase?
TF6215B74145	Elongation factor
TF6215B6553	Granule bound starch synthase
P51WP57134	Heat shock factor?
P51WP4847	phosphatase? Phosphomannomutase?
P45NP51144	Cysteine protease? Glutathione transferase? Xyloglucan endo-transglycosylase?
P45NP31207	Glucose-1-phosphate adenylyltransferase? Subtilisin-chymotrypsin inhibitor?
P45NP21816	Chloroplast 50 S ribosomal protein
GPB49459	Pyruvate dehydrogenase kinase
GPB41819	rust resistance gene? Hordein?
GPB5902	potassium transporter
GPB16764	disease resitance gene
GPB1614	Peptidyl-prolyl cis-trans isomerase
GPB25359	ABC transporter related?
GPB17570	Histidine acid phosphatase?
GPB26526	Myb-related protein
GPB320	Calmodulin-like protein? Inositol 1,3,4,5,6-pentakisphosphate 2-kinase?L-ascorbate oxidase precursor?DNA-directed RNA polymerase?
P45NP20958	Phenylalanine ammonia-lyase
GPA42571	Ribulose bisphosphate carboxylase

### Experimental validation of the approach

**Figure 5:**
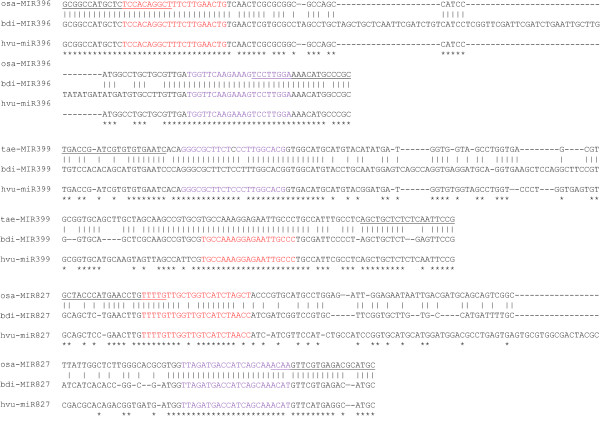
**Sequence alignment of each of pre-miRNA396, pre-miRNA399 and pre-miRNA827 from rice, Brachypodium and/or wheat**. Sequences of the miRNAs and miRNA*s are highlighted in red and purple, respectively. The underlined sequences correspond to the primers used for the PCRs as described in the text.

Our search for barley miRNAs used genomic sequences of rice, Brachypodium and wheat because the number of available genomic barley sequences is limited. To test the validity of this approach, we first selected three conserved miRNAs, namely miR396, miR399 and miR827. miR399 and miR827 are both important for plant uptake of phosphorus under phosphorus deficiency [62], while miR396 is important in controlling cell proliferation [63]. None of the corresponding pre-miRNAs are present in the limited available barley genomic sequence. To identify pre-miR396, pre-miR399 and pre-miR827 in barley, sequences of pre-miRNA sequences from rice, Brachypodium and/or wheat were aligned to determine conserved regions (Figure 5). A PCR primer pair was then designed to the conserved regions for each (all the primer sequences used for the PCR are underlined in Figure 5) in order to maximize the chance that it would be suitable for amplification of the corresponding barley DNA. PCR amplification was carried out using barley genomic DNA with the specific primer pair. In each case, specific PCR products of the expected size were produced. Sequencing of these PCR products showed high sequence similarity of the barley pre-miRNAs with those from rice, Brachypodium or wheat (Figure 5), confirming that they are indeed barley pre-miR396, pre-miR399 and pre-miR827.

To further confirm the presence of pre-miRNA transcripts in barley, RT-PCR was applied using the primer pairs corresponding to each of the miRNA and miRNA* sequences. Fragments of expected sizes were obtained and sequenced. The RT-PCR products were identical in sequence to the above PCR products (data not shown). Hence, these results confirm actual expression of pre-miR396, pre-miR399 and pre-miR827.

**Figure 6:**
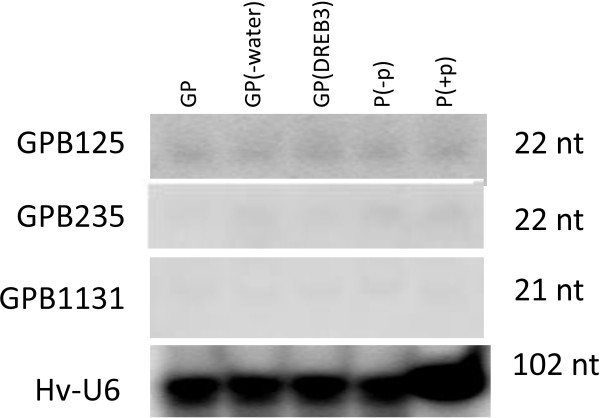
**Northern blot detection of three novel miRNAs, GPB125, GPB235 and GPB1131, from barley leaf tissues**. U6 was used as loading control. Sizes of each miRNA and U6 are indicated. Uppercase P and GP represent the Pallas cultivar and the Golden Promise cultivar, respectively. GP also indicates Golden Promise plants grown under well-watered conditions. P (-p) represents Pallas plants grown in soil supplied with 22.5 mg phosphate (KH_2_PO_4_)/kg dry soil, while P (+p) represents Pallas plants grown in soil supplied with 75 mg phosphate (KH_2_PO_4_)/kg dry soil. GP (DREB3) represents Golden Promise plants transformed with the wheat DREB3 gene. GP (-water) represents Golden Promise plants grown under drought conditions (see Methods for details).

Next, we validated the predicted novel miRNA candidates in barley using Northern hybridization and RT-PCR. Three miRNA candidates (GPB125, GPB235 and GPB1131) were chosen for the following reasons: 1) they were relatively abundant (Table 6), thereby enabling detection, especially with the low sensitivity of Northern hybridisation, and 2) they all have detected miRNA* sequences (Table 6), hence allowing the use of two specific primers in RT-PCR to increase the specificity of amplification. All three miRNA candidates were detected by Northern hybridization as discrete bands sized around 21 nt (Figure 6). U6 snRNA was used as a loading control because this RNA is stably expressed at high abundance under different biological conditions. RT-PCR was performed using primers corresponding to part of the miRNA and miRNA* sequences (see the underlined sequences in Table 6). The result showed that all primer pairs generated small RT-PCR products sized between 70 nt and 100 nt. Cloning and sequencing of these products showed that they contained the correct miRNA and miRNA* sequences, indicating that they are most likely the pre-miRNAs of GPB125, GPB235 and GPB1131. As the used PCR primers overlapped with part of the miRNA and miRNA* sequences, it is impossible to know whether multiple variants were present.

## Discussion

This work provides a comprehensive study of the miRNA content of the barley transcriptome and we hope that it serves as a foundation for future, more targeted, studies of individual barley miRNAs and their function. The existence of associated miRNA-like hairpin structures in rice, wheat, Brachypodium or barley was taken to be necessary, but not sufficient, evidence for the interpretation of a sequencing read as a potential mature miRNA. The rice and Brachypodium genomes have been fully sequenced, while the wheat and barley genomes have not. Consequently, it was more likely for us to find hairpin-like structures in the data for the former two species. Indeed, for those 44 miRNA candidates not already identified in other species, 35 showed evidence for a hairpin precursor in Brachypodium and/or rice, but not wheat or barley. Evidence for only 6 miRNA candidates came from available barley sequence. While this may at first appear paradoxical, there are several plausible explanations. It is possible that these hairpins give rise to expressed miRNA in these other species as well as barley but that this expression has, so far, not been observed. Indeed, this may very well be the most likely explanation as the total number of reads in our study exceeds those of other comparable works by an order of magnitude or more. One would expect, therefore, increased sensitivity to weakly expressed miRNA in the present work. Most, but not all, of our novel candidates are indeed expressed at relatively low levels. Alternatively, species-specific changes in transcriptional regulation may have caused a down-regulation of expression of these miRNA in the other grasses or, indeed, they may have become non-functional.

While our large dataset enabled discovery of weakly expressed miRNA, it also drew attention to the interplay between total read number (i.e. 'sequencing depth') and sequencing error if the genome sequence of the species under consideration is not known. In a sequencing experiment of genomic DNA, sequencing error can largely be accounted for by filtering based on read quality and/or the imposition of a suitable lower limit in read depth. In this type of experiment, increasing the read number generally provides increased accuracy of results, even in the absence of decreased sequencing error. In a transcriptome sequencing experiment such as ours, on the other hand, the read depth itself is of inherent interest as it provides an indication of the level of transcription. For this type of experiment, where both highly as well as weakly expressed transcripts are important, increased accuracy is obtained by decreasing *the product *of the total read number and sequencing error. Filtering based on read quality is not sufficient per se, which caused us to make an assessment of the reliability of a read based on its abundance relative to the abundance of any other reads with highly related sequences. A natural consequence is that it is difficult to reliably detect, in the presence of a highly abundant miRNA, additional members of the same family unless they are themselves sufficiently abundant.

Furthermore, analysis of read abundances of sequence variants allowed an assessment of the sequencing error rates independent of those supplied in sequence quality files. This is important as Dohm et al. [64] found that the error rate provided by the Solexa platform may underestimate the true error by a factor of up to 100 for the highest quality reads. For our dataset, the provided scores appear, on average, to underestimate the true sequencing error by a factor of two or three. Furthermore, we see some indication of correlations in the occurrence of multiple read errors. Such correlations were also noted by Dohm et al. [64]. However, it was beyond the scope of the present work to investigate these further.

We compared the barley miRNA candidates with those known to be expressed in rice, wheat or Brachypodium. However, a definitive comparison is difficult because of differing, and often somewhat arbitrary, criteria used to support miRNA discovery. For example, our filtering of repetitive sequences is more stringent than that used by other researchers summarised in Table 1 and, in any case, the repeat databases used for this filtering are in a constant state of flux, with new sequences continually being added. Hence, some published miRNA sequences could well be classified as 'repetitive sequence' in one study, but not in another. Furthermore, there are small differences in the literature, as well as our own work, in the assessment on whether a given putative pre-miRNA sequence admits a valid folded hairpin structure. Similarly, many of the differences in our findings to those of computational searches of barley EST-library published recently [28] can be explained by differing definitions of what constitutes a 'known' miRNA, filtering of repetitive sequences and sequences not included in the relevant version of miRBase [28]. The most notable difference that remains is that, as opposed to [28], we do not see any evidence of expression of some of the homologs of the rice miRNAs described as novel by Zhu et al [17], i.e. miR1848, miR1858, miR1862, miR1867 and miR1871. While it is quite possible that these miRNAs are simply not expressed in leaf, the only tissue probed in our study, the studies listed in Table 1 indicate that they have also not been found to be expressed in wheat and Brachypodium. On the other hand, we see evidence of strong expression of miR167, miR529, miR1318/1432, while these were not detected in [28]. These sorts of differences between studies serve as a reminder that, without direct confirmation of target degradation, many predicted and putatively expressed miRNAs should be viewed as candidates only.

We found the read depth distribution along putative pre-miRNAs to be a reliable guide for differentiating possible miRNAs from contaminant sequences such as degradation products of mRNAs or transcripts simultaneously expressed in both sense and antisense orientations. Known miRNAs were invariably characterized by sharply defined distributions, while mRNAs exhibited degradation products spread much more uniformly along the parental sequence.

A further possible source of contamination is transcription of ncRNAs from the chloroplastic genome. While those reads matching known chloroplastic tRNA and rRNA sequence were filtered out, we did note that a disproportionate number of reads in our original dataset were identical to known non-coding chloroplastic sequence. Specifically, while the length of the known non-coding regions of the chloroplast genome of barley is only about 0.002% of the anticipated length of its nuclear genome, over 0.7% of the ~3.7 million unique reads in our dataset could be located on these portions, i.e. a rate that is over 400 times that expected if these regions were transcribed at the same rate in the two genomes. Indeed, we found reads matching a little more than 80% of the entire chloroplast genome, in both orientations, in our dataset. We did not find any evidence for preferred simultaneous sense and antisense transcription since we found that roughly 65% of the chloroplast is simultaneously transcribed in both orientations. Abundant transcription of ncRNAs from intergenic chloroplastic regions has also been noted elsewhere [65]. These small noncoding RNAs may play an important role in the regulation of chloroplast genes [65]. The existing antisense RNAs could regulate RNA stability in chloroplasts [66]. We did not explicitly eliminate the reads that are associated with the chloroplastic sequences as these could also come from the nuclear genome [15]. Nevertheless, we did not find any reads that originated from chloroplastic sequence able to form a valid pre-miRNA hairpin structure.

Finally, while the present study represents a beginning, a true characterization of the repertoire of miRNA expression in barley will only emerge through additional experiments sampling expression across different tissues, different developmental stages and/or environmental conditions (e.g. imposed stresses). Furthermore, it will also be greatly aided by the completion of the barley genome sequence, which will enable direct identification of precursor miRNA sequences within barley directly, rather than relying on those present in related species. Matching of reads to the genome will also reduce or eliminate the need for sequence quality filtering, thereby enhancing one's capacity to detect closely related variants. It should also allow the discovery of multiple precursor sequences for individual, identical, mature miRNAs. Within the present study this is not possible but, judging by the abundance of such cases found in the rice genome, one can expect them to contribute significantly to the full complement of miRNAs encoded in the barley genome.

## Conclusion

We have shown that deep sequencing of small RNAs can provide a powerful tool for miRNA discovery, even for species whose genome has not been sequenced. While deep sequencing datasets can be extremely large, we demonstrate that even in the absence of a genome sequence suitable read filtering tools can be designed for isolating the relatively small miRNA component. Using these tools, we have explored the expression of miRNAs in barley leaves. Of the 100 miRNAs identified, roughly half have orthologs known to be expressed in other grass species, while the remainder appear to be specifically expressed in barley. Our study provides the first large scale view of miRNAs in barley and will help to elucidate the roles of miRNAs in this and other cereal crops.

## Methods

### Plant materials

The barley (Hv) cultivars, Golden Promise and Pallas, as well as a genotype of Golden Promise transformed with the wheat *DREB3 *gene, were used for the analysis of small RNAs in barley. Pallas plants were grown in soil supplied with either 22.5 mg or 75 mg phosphate (KH_2_PO_4_)/kg dry soil. Basal nutrients and calcium carbonate were added into the soil, and growth conditions were the same as those described by Genc et al. [67]. The Pallas plants were harvested 16 days after seed imbibitions.

The Golden Promise plants were grown in 6 inch pots in coco-peat soil in a glasshouse at 22-23°C day/16°C night, with a 12-hour day/night light cycle for 3 weeks under well-watered conditions. Leaves were then sampled from both transformed and untransformed plants. Untransformed Golden Promise plants were grown for another 5 days without watering and more leaves were sampled for RNA isolation.

### Total RNA isolation

Total RNA was isolated from leaves using TRIzol (Invitrogen, Carlsbad, CA, USA) according to the manufacturer's instructions. Briefly, 0.5 g leaf material was ground to a fine powder in liquid nitrogen and total RNA was extracted using 2.4 ml TRIzol reagent and 500 μl chloroform, and precipitated with 2 ml isopropanol. The precipitated RNA was washed once with 700 μl 75% ethanol and re-suspended in 100 ìl DEPC-treated water. In this way, approximately 100 μg of total RNA was obtained for each sample.

### Small RNA isolation and sequencing

Low molecular weight RNA was isolated using the Purelink miRNA isolation kit (Invitrogen, Carlsbad, CA, USA) according to the manufacturer's instructions and then further purified as follows. RNA was size fractionized by electrophoresis on 15% polyacrylamide (30:0.8) gels containing 7 M urea in TBE buffer (45 mM Tris-borate, pH 8.0, and 1.0 mM EDTA). The RNA fraction of 18 nt to 30 nt in size was excised from the gel and recovered in 0.3 M NaCl at 4°C overnight. The recovered RNAs were precipitated with isopropanol containing 5 μg/mL glycogen, and ligated to a 5' adaptor (5'-GUUCAGAGUUCUACAGUCCGACGAUC-3') using T4 RNA ligase. The ligated RNAs were size fractionated on a 15% polyacrylamide gel containing 7 M urea and then eluted and precipitated as above. The recovered RNAs were ligated to a 3' adaptor (5'- pUCGUAUGCCGUCUUCUGCUUGUidT-3', p, phosphate, idT, inverted deoxythymidine) using T4 RNA ligase, followed by another size fractionation. After recovery, the ligated RNAs were reverse transcribed and amplified as described in Illumina's small RNA preparation protocol. The amplified cDNA products obtained from the five samples were sequenced in separate lanes using the 36-base Illumina platform.

### Genomic DNA Isolation and amplification

Barley genomic DNA isolation was achieved using a DNA mini-prep method adapted from Rogowsky et al. [68]. DNA extraction buffer (100 mM Tris, 100 mM NaCl, 10 mM EDTA, 1% Sarkosyl, 0.1% Na_2_SO_3_, 2% insoluble Polyvinypolypyrrolidone [pH 8.5]) and phenol-chloroform-isoamyl alcohol (25:24:1) were used.

Amplification was performed using the following condition: 7 cycles at 94°C for 25 seconds (sec) and 72°C for 1 minute (min), followed by 32 cycles at 94°C for 25 sec and 67°C for 1 min. PCR products were analysed on a 2% (w/v) agarose/Ethidum bromide gel along with 100 bp DNA size marker.

### Northern blot hybridization

50 μg of total RNA (prepared as described above) was separated on a 15% polyacrylamide gel containing 7 M urea. The electrophoresis was stopped when the bromophenol blue dye reached the bottom of the gel, and RNAs were transferred to Hybond-N membrane (Amersham Bioscience) using 20 × SSC. After transfer, RNA was fixed to the membrane by UV-crosslinking and subsequent heating at 80°C for two hours. Blots were cut in the middle. The top piece was hybridized with a ^32^P labelled DNA oligonucleotide probe complementary U6 sequence, made by end-labelling with γ-^32^P-ATP using T4 polynucleotide kinase (New England Biolabs, Beverly, MA). U6 served as a loading control. The bottom piece was hybridized with γ-^32^P labelled oligonucleotide probes complementary to predicted miRNA sequences. All blots were pre-hybridized and hybridized at 37°C in 125 mM Na_2_HPO_4 _(pH 7.2), 250 mM NaCl_2_, 7% SDS, and 50% formamide, and washed at 37°C twice with 2 × SSC, 0.2% SDS, followed by a higher stringency wash of 1 × SSC, 0.1% SDS at 37°C if required. Hybridization signals were visualised using a Phophorimager (Typhoon Trio, Amersham Bioscience).

### RT-PCR

The first strand cDNA was synthesized using total RNA as described above. The RT reaction was performed using SuperScript III Reverse Transciptase (Invitrogen) in an automated thermocycler (PTC-100, MJ Research) by incubation at 50°C for 1 hour followed by 15 min at 70°C to inactivate the reaction. Amplification was carried out by heating at 95°C for 7 min, and then 40 cycles of incubation at 94°C (10 sec), 60°C (30 sec) and 68°C (30 sec), with a final extension step of incubation at 68°C for 5 min. The amplified DNA fragments were electrophoresed on a 2% (w/v) agarose gel.

### DNA purification, cloning and sequencing

DNA fragments were separated in a 2% 1 × TAE buffered agarose gel, extracted using NucleoTrap (Macherey-Nagel) and cloned into pGEM-T Easy vector (Promega). Sequencing was carried out with T7 and SP6 primers using a 96-capillary 3730*xl *DNA analyzer (Applied Biosystems).

### Bioinformatic analysis

The sequencing runs resulted in over 34 million reads distributed roughly equally among the five samples. Sequences with homopolymer runs longer than 6 bases, mostly consisting of A's, were removed. Adapters were trimmed off the 36 mer reads using a stepwise procedure in order to allow for sequencing errors. Specifically, the 5' adapter was removed starting by searching for the 17 rightmost bases, allowing 4 mismatches and finishing with the 10 rightmost bases, not allowing for any mismatches. Similarly, the 3' adapter was removed by searching for the 22 leftmost bases, allowing 8 mismatches and finishing with the 7 leftmost bases, allowing for one mismatch. After truncation, sequences with a length shorter than 18 bases or equal to 36 bases were removed. This resulted in a dataset of almost 29 million reads (~3.7 million unique reads). Not unexpectedly, only ~218 000 of these unique reads match the limited available genomic barley BAC sequences. Those reads that do not match are expected to either be sequencing errors or are located on parts of the genome not covered by these BACs. Similarly, only ~339 000 of the unique reads match the available EST-derived barley tentative contigs (HVGI Release 10), no doubt representing an underestimate of the total number of reads matching genic regions as the available barley EST collections are not complete.

### Culling of reads with potential sequencing errors

One expects the relative abundance profiles of parent and variant sequences to be given by a multinomial distribution. If the probability *p *of a single base-error *N→N' *is small and if, for simplicity, we take this probability to be independent of *N *and base position, it is straightforward to estimate that the expected number of distinct 1-SNP variants *Y *of an abundant parental sequence of length *L *and abundance *X *is closely approximated by

$$Y=3L[1-\;{\left(1-p\right)}^{X}].$$

This implies that for very abundant reads (the most abundant in our dataset has close to 10^7 ^copies) every single 1-SNP variant is present in significant quantities, and most of the 9L (L-1)/2 possible 2-SNP variants should also be present. The above functional form agrees well with the data shown in Figure 2 for a value of p ~ 0.0007, which corresponds to a per-read error rate of about 4%. Direct examination of the quality information provided by the Solexa sequencer leads to an estimate for the average error of p ~ 0.00025 for this dataset, i.e. a factor of 2 or 3 lower than that actually seen in Figure 2. Note also that filtering of the dataset by supplied quality scores is not sufficient. The highest quality reads are the ones on the right hand side of the plotted band in Figure 2 and, clearly, still contain an unacceptable proliferation of technical variants.

Motivated by these considerations, we elected not to make use of the Solexa quality scores in filtering our dataset. Rather, we took the following approach: any parent sequence in the dataset with abundance *X *can be expected to be accompanied, on average, by 1-SNP variants of abundance *pX*. Hence, we cull variants based on their abundance: if they are very abundant (i.e. abundance >>*pX*), the variant is likely to be a true biological variant and is kept, while if the variant has an abundance of roughly *pX *or less, it is consistent with being a technical variant and is removed. More precisely, erring on the conservative side in order to allow for statistical fluctuations, we remove those reads with an abundance of less than 0.12% of the most abundant possible parent sequence. Furthermore, we also culled any reads with an abundance of less than 12. This removes the majority of higher-SNP variants still present. This filtering procedure, based on relative abundances of sequence variants rather than quality information alone, permits the discovery of multiple closely related miRNA family members, unless their relative abundances are consistent with them being technical sequence variants of each other.

### Culling of repetitive sequences, rRNA, tRNA etc

All sequences were compared, using Blast [69] as well as rmapper V1.10 from the SHRiMP software package (http://compbio.cs.toronto.edu/shrimp/,[70]), against a collection of cereal rRNA, tRNA and snoRNA databases at NCBI and Rfam in order to eliminate known contaminating RNA sequences, wherever possible erring on the conservative side. Sequences which matched to these RNA collections either perfectly or with one mismatch to a barley or wheat sequence, or with up to three mismatches to a rice- or other sequence, were removed from the dataset. Alltogether, this removed ~23500 reads matching rRNAs and ~4300 matching tRNAs from our set of unique sequences. Furthermore, sequences that matched to the Triticeae Repeat Database TREP, obtained from Graingenes http://www.graingenes.org, again allowing for mismatches, were also removed.

### Presence of hairpin

All remaining sequences were mapped to a selection of genomic sequences to determine whether there is evidence for an associated hairpin structure characteristic of miRNAs, either in barley or in related genomes. For this purpose we used barley and wheat BAC sequences downloaded from NCBI, the HVGI gene indices (http://compbio.dfci.harvard.edu, V10) for barley as well as the rice genome (http://rice.plantbiology.msu.edu, V6.0) and the Brachypodium draft genome (http://www.brachypodium.org/, 4 × checkpoint assembly). We allowed up to 3 mismatches (or 80% of the maximum score, whichever provided the tighter bound) when mapping to rice or Brachypodium, one mismatch when comparing to wheat and no mismatches when comparing to barley sequence. Mapping was performed using rmapper. Genomic regions around the mapped sites were used to predict the energetically most favourable RNA secondary structures by making use of the Vienna RNA package (V 1.8.1, http://www.tbi.univie.ac.at/~ivo/RNA/). These structures were then evaluated for suitability as plant miRNA precursors using the MIRcheck software package and parameters as described in Jones-Rhoades and Bartel [19].

### Read clustering and analysis of read distributions

Reads were flagged as possible miRNAs if MIRcheck indicated any one of the rice, Brachypodium, wheat or barley genomic sequences to give rise to an acceptable miRNA precursor secondary structure. Because of the tolerance of mismatches to non-barley genomes and the presence of miRNA families, remaining technical errors associated with sequencing and the ligation of adapters, as well as biological variation arising from imprecise excision of the miRNA/miRNA* duplex by DCL1, usually groups of these reads were associated with candidates for individual miRNAs. For this reason, reads that passed MIRcheck were clustered together transitively if the Smith-Waterman similarity between two reads was greater than 60% of its maximum attainable value. This resulted in 429 read clusters, each one associated with a set of acceptable miRNA precursor sequences and each cluster potentially corresponding to a miRNA family. These clusters were merged further if the associated genomic sequences were overlapping or immediately adjacent to each other.

Because of the well-known propensity of RNA to fold [71], we expected this set of clusters to contain, as well as miRNAs, degradation products of expressed mRNA as well as other non-miRNA sequences. In the absence of a sequenced barley genome, the gene content of this species is only partially known. For this reason, we did not rely on the barley tentative contigs to screen out reads originating from coding RNA. Rather, we made use of the very high sequencing depth of our study, which enabled a direct classification of read distribution patterns associated with our putative miRNA precursors. First, we mapped all reads in our dataset, allowing for up to three mismatches, to all miRNA precursors associated with the 429 read clusters. Some of these clusters correspond to well-known plant miRNAs and we found that for all of these cases the read distribution is very distinctive in that a multitude of different reads, often of very high abundance, were associated with the mature miRNA and the miRNA* regions of the precursor miRNA, but virtually no reads aligned to the intervening region. An example of such a read distribution is shown in Figure 3. We surmise from this that the excised portion of the miRNA hairpin is degraded very rapidly, at least in plants where this degradation takes place in the nucleus [2]. This precise excision of the miRNA/miRNA* duplex is deemed the 'sole criterion that is both necessary and sufficient for miRNA annotation' defined by Meyers et al. [39] in their guidelines for plant miRNA annotation. We found Figure 3 to be typical of most known rice miRNAs for which there is evidence of expression of barley counterparts, the only variation on the theme being those miRNAs with multiple mature miRNAs per precursor. The latter were, however, readily distinguishable.

Degradation products of mRNA, on the other hand, did not show this double-peaked read-profile. Rather, for coding RNA we found a very distinctive read profile extending throughout the aligned region, with no clear abundance peaks. An example of such an alignment is shown in Additional file 5. Similarly, some read clusters could be excluded from further analysis as they showed evidence for transcription both in the plus/plus and plus/minus orientation. This sense-antisense transcription was not seen for any of the miRNA candidates with known homologs in rice. Finally, evidence for substantial open reading frames was also used to exclude a limited number of clusters. In summary, we ended up with 63 clusters that were classified as putative miRNAs or miRNA families.

## Authors' contributions

AS and UB carried out the bioinformatics analysis and drafted the manuscript. BS participated in the design of the study, performed the experiments and drafted the manuscript. CH participated in the sequence alignment and experiments, and helped to draft the manuscript. PL conceived of the study, participated in its design, coordination and helped to draft the manuscript. All authors read and approved the final manuscript.

## Supplementary Material

Additional file 1 **Candidates for barley miRNAs previously described in rice, Brachypodium or wheat**. Click here for file

Additional file 2 **Precursors for 'barley-specific' miRNAs**. Click here for file

Additional file 3 **Secondary structure of putative hairpin orthologs for the barley miRNA candidates listed in Table **6. Only the most compact hairpin in the species most closely related to barley is shown and annotated with species, chromosome/accession ID, orientation and location of the match (Notation: osa and bdi-rice and Brachypodium genome, respectively; Ta and Hv BAC-wheat and barley BAC sequences, respectively; Hv TC-barley tentative contigs). The location of the approximate match of the barley miRNA candidate on the hairpin is indicated by a solid bar. Click here for file

Additional file 4 **Target candidates for putative miRNAs**. Click here for file

Additional file 5 **Typical alignment of reads to a putative precursor region that does not support interpretation as a miRNA**. Most likely this kind of alignment profile is indicative of reads originating from degraded mRNA. This particular graphic shows the alignment of reads to a portion of the *Triticum aestivum *BAC sequence AC200830. Click here for file
