# Supplementary material for: Discovery of barley miRNAs through deep sequencing of short reads
Source: BMC Genomics. 2011 Feb 25;12:129. doi: 10.1186/1471-2164-12-129 (PMC3060140; doi:10.1186/1471-2164-12-129)
Supplement: Additional file 1 — Candidates for barley miRNAs previously described in rice, Brachypodium or wheat. [file 1471-2164-12-129-S1.DOCX]

**Additional file 1.** Candidates for barley miRNAs previously described in rice, Brachypodium or wheat.

| miRNA  family | Read name^a^ | Proposed  miRBase name | Read sequence | Read Length | Abundance | Relationship to known miRNAs^b^ | | |
| --- | --- | --- | --- | --- | --- | --- | --- | --- |
|  |  |  |  |  |  | Rice | Brachypodium^c^ | Wheat^d^ |
| miR156/  miR157 | P51WP2 | miR156a | UGACAGAAGAGAGUGAGCAC | 20 | 1060913 | miR156(a-j) | miR156d | miR156a |
|  | GPA134 | miR156d | UGACAGAAGAGAGUGAGCAU | 20 | 9977 |  |  | (07)-miR156m [1] |
|  | TF6215B422^R^ | miR156b | UGACAGAAGAGAGCGAGCAC | 20 | 3537 |  | miR156b |  |
|  | P51WP683^R^ | miR156c | UGACAGAAGAGAGGGAGCAC | 20 | 1987 |  | miR156c |  |
|  |  |  |  |  |  |  |  |  |
| miR159/  miR319 | TF6215B321 | miR159 | UUUGGAUUGAAGGGAGCUCUG | 21 | 4887 | miR159(a,b) | miR159c | (07)-miR159(a,b), (09)-miR159c |
|  | P45NP25333 | miR319 | CUUGGACUGAAGGGUGCUCCCU | 22 | 25 |  | miR319(b,h) [1] |  |
|  |  |  |  |  |  |  |  |  |
| miR160 | GPA1649 | miR160a | UGCCUGGCUCCCUGUAUGCCA | 21 | 553 | miR160(a-d) | miR160c | (09)-miR160, (09)-miR160a |
|  | P45NP3824 | miR160b | UGCCUGGCUCCCUGAAUGCCA | 21 | 234 | miR160f | miR160a | (07)-miR160a, (09)-miR160b |
|  |  |  |  |  |  |  |  |  |
| miR164 | P51WP57 | miR164a | UGGAGAAGCAGGGCACGUGCA | 21 | 18651 | miR164(a,b,f) | miR164a | miR164a |
|  | P51WP10424 | miR164b | UGGAGAAGCAGGGCACGGGCA | 21 | 143 | miR164e [3] |  |  |
|  | P51WP39877 | miR164c | UGGAGAAGCAGGGCACGUGAA | 21 | 25 | miR164e [1] |  |  |
|  | P45NP232^R^ | miR164d | UGGAGAAGCAGGGCACGUGCU | 21 | 5116 | miR164d | miR164b | (09)-miR164b |
|  |  |  |  |  |  |  |  |  |
| miR165/  miR166 | P51WP14 | miR166b | UCGGACCAGGCUUCAUUCCCC | 21 | 89352 | miR166(a-d,f,n) | miR166c, (U)-miR165a | (09)-miR166a |
|  | P51WP25 | miR166c | UCGGACCAGGCUUCAAUCCCU | 21 | 39515 | miR166(k,l) | miR166a | (09)-miR166b |
|  | P51WP186 | miR166d | UCGGACCAGGCUUCAUUCCUU | 21 | 4639 |  | miR166g | (09)-miR166c |
|  | P51WP2959 | miR165 | UCGGACCAGGCUUCAUCCCCC | 21 | 151 |  | miR165 | (09)-miR165 |
|  |  |  |  |  |  |  |  |  |
| miR167 | P45NP17 | miR167d | UGAAGCUGCCAGCAUGAUCUGA | 22 | 119085 |  |  | (07)-miR167m |
|  | P51WP60 | miR167a | UGAAGCUGCCAGCAUGAUCUA | 21 | 14651 | miR(167a-c) | miR167a, (U)-miR167 | miR167a |
|  | GPB213 | miR167b | UGAAGCUGCCAGCAUGAUCUGC | 22 | 6213 |  | miR167e [1] |  |
|  | P51WP5353 | miR167c | UGAAGCUGCCAGCAUGAUCUC | 21 | 97 |  | miR167c |  |
|  |  |  |  |  |  |  |  |  |
| miR168 | P45NP1 | miR168a | UCGCUUGGUGCAGAUCGGGAC | 21 | 1921554 | miR168a | miR168c | miR168a |
|  | P51WP147 | miR168b | UCGCUUGGUGCAGCUCGGGAC | 21 | 6414 |  |  | (07)-miR168b [2] |
|  | P51WP151 | miR168c | UCGCUUGGUGCAGAUCGGGCC | 21 | 5346 |  |  | (09)-miR168b [3] |
|  | GPB390 | miR168d | UCGCUUGGUGCAGAUCGGGAU | 21 | 4826 |  |  | (07)-miR168b |
|  | GPA5105 | miR168e | UCGCUUGGGCAGAUCGGGAC | 20 | 108 |  | miR168a |  |
|  | P51WP106277 | miR168f | UCGCUUGGUGCAGCUCGGGAA | 21 | 15 | miR168b [2] |  |  |
|  |  |  |  |  |  |  |  |  |
| miR169 | P45NP1509 | miR169e | UAGCCAAGGAUGACUUGCCG | 20 | 585 |  | miR169f |  |
|  | GPB2134 | miR169a | CAGCCAAGGAUGACUUGCCGA | 21 | 482 | miR169a | miR169a,(U)-miR169a | miR169a |
|  | GPB6437 | miR169c | UAGCCAAGAAUGACUUGCCUA | 21 | 156 | miR169(n,o) | miR169c | (09)-miR169c |
|  | P51WP5129 | miR169d | UAGCCAAGGAUGACUUGCCUG | 21 | 148 | miR169(h-m) | miR169i | (07)-miR169m, (09)-miR169h |
|  | P45NP20061 | miR169f | UAGCCAAGGAUGAUUUGCCUGUG | 23 | 39 |  |  | (09)-miR169i |
|  | P51WP124^R^ | miR169b | CAGCCAAGGAUGACUUGCCGG | 21 | 7887 | miR169(b,c) | miR169b | miR169b |
|  |  |  |  |  |  |  |  |  |
| miR170/  miR171 | P45NP1952 | miR171 | UGAUUGAGCCGUGCCAAUAUC | 21 | 659 | miR171(b-f) | miR171a, (U)-miR170 | (07)-miR171a, (09)-miR171e |
|  | P45NP6428 | miR171a | UGAUUGAGCCGCGCCAAUAUC | 21 | 124 | miR171a | (U)-miR171(a,d) | (09)-miR171d |
|  |  |  |  |  |  |  |  |  |
| miR172 | GPB36^R^ | miR172a | AGAAUCUUGAUGAUGCUGCAU | 21 | 40700 | miR172(a,d) | miR172c | (07)-miR172a, ta(09)-miR172b |
|  | GPB5616^R^ | miR172b | AGAAUCUUGAUGAUGCUGCCU | 21 | 210 |  | miR172b [2] |  |
|  |  |  |  |  |  |  |  |  |
| miR390 | P51WP689 | miR390 | AAGCUCAGGAGGGAUAGCGCC | 21 | 1118 | miR390 | miR390 | miR390 |
|  |  |  |  |  |  |  |  |  |
| miR393 | GPB62 | miR393 | UUCCAAAGGGAUCGCAUUGAU | 21 | 20621 | miR393 [2] | miR393a [2] | (07)-miR393 [2], (09)-miR393a [2] |
|  |  |  |  |  |  |  |  |  |
| miR394 | P51WP5467^R^ | miR394 | UUGGCAUUCUGUCCACCUCC | 20 | 115 | miR394 | miR394 |  |
|  |  |  |  |  |  |  |  |  |
| miR395 | TF6215B1877 | miR395 | UGAAGUGUUUGGGGGAACUC | 20 | 323 |  | miR395b | (09)-miR395b |
|  |  |  |  |  |  |  |  |  |
| miR396 | P45NP122 | miR396d | UCCACAGGCUUUCUUGAACUG | 21 | 13424 | miR396(d,e) | miR396a | (09)-miR396a |
|  | P51WP12310 | miR396b | UCCACAGGCUUUCUUUAACUG | 21 | 143 | miR396(a,b) [3] | miR396d [3] | (07)-miR396a [3], (09)-miR396d [3] |
|  | P45NP10536 | miR396a | UUCCACAGCUUUCUUGAACUG | 21 | 78 | miR396(a,b) | miR396d | (07)-miR396a, (09)-miR396d |
|  | P45NP1558^R^ | miR396c | UUCCACAGCUUUCUUGAACUU | 21 | 414 | miR396c | miR396e | (09)-miR396e |
|  |  |  |  |  |  |  |  |  |
| miR397/  miR2029/  miR2508 | P45NP5920 | miR397a | UUGAGUGCAGCGUUGAUGAAC | 21 | 108 |  | miR397c;  miR2508 [1] | (09)-miR2029 |
|  |  |  |  |  |  |  |  |  |
| miR399 | P45NP1202 | miR399c | UGCCAAAGGAGAUUUGCCCAG | 21 | 231 | miR399(e-g) |  |  |
|  | P45NP1814 | miR399d | UGCCAAAGGAGAGUUGCCCUG | 21 | 195 | miR399d | miR399c |  |
|  | P45NP1994 | miR399b | UGCCAAAGGAGAAUUGCCCUG | 21 | 154 | miR399(a-c) | miR399b | (09)-miR399 |
|  | P45NP2143 | miR399 | UGCCAAAGGAGAUUUGCCCCG | 21 | 134 | miR399k [1] | miR399a [1] |  |
|  |  |  |  |  |  |  |  |  |
| miR408 | P51WP9508^R^ | miR408 | UGCACUGCCUCUUCCCUGGC | 20 | 48 | miR408 [1] | miR408 [1] | (07)-miR408 [1] |
|  |  |  |  |  |  |  |  |  |
| miR444/  miR2024 | P45NP21798 | miR444c | UGCAGUUGUUGUCUCAAGCUU | 21 | 49 | miR444(b.2,c.2) | miR444c |  |
|  | P51WP1729^R,NH^ | miR444d | UGUUGUCUCAAGCUUGCUGCC | 21 | 708 | miR444(b.1,c.1) | miR444d | (09)-miR444a |
|  | GPB3214^R^ | miR444a | UGCAGUUGCUGCCUCAAGCUU | 21 | 339 | miR444(a.2,d.2,e) | miR444b | (09)-miR444b, (09)-miR2024b |
|  | P51WP5346^R^ | miR444b | UGCAGUUGCUGUCUCAAGCUU | 21 | 233 |  |  | (09)-miR444c, (09)-miR2024a |
|  | GPB100121^R^ | miR444a | UUGUGGCUUUCUUGCAAGUCG | 21 | 15 | miR444d.3 [1] | miR444a [1] |  |
|  |  |  |  |  |  |  |  |  |
| miR516 | GPA1606^R^ | miR516 | AGCAAGGAUUGACAGACUGA | 20 | 642 |  |  | (07)-miR516 [3] |
|  |  |  |  |  |  |  |  |  |
| miR528 | P51WP34 | miR528 | UGGAAGGGGCAUGCAGAGGAG | 21 | 18175 | miR528 | miR528 | (09)-miR528 |
|  |  |  |  |  |  |  |  |  |
| miR530 | GPA18451^R^ | miR530 | CUGCAUUUGCACCUGCACCUA | 21 | 31 | miR530-5p [1] |  |  |
|  |  |  |  |  |  |  |  |  |
| miR531 | GPA16004^R,NH^ | miR531 | CUCGGCGGGGCAGCGUGCAG | 20 | 37 | miR531b [3] |  |  |
|  |  |  |  |  |  |  |  |  |
| miR827 | P45NP39 | mir827 | UUAGAUGACCAUCAGCAAACA | 21 | 12497 | mir827 [3] |  |  |
|  |  |  |  |  |  |  |  |  |
| miR1126 | P45NP8277^R^ | miR1126 | UUACACUAUGGACUAGAUACGGAG | 24 | 48 |  |  | (07)-miR1126 [3] |
|  |  |  |  |  |  |  |  |  |
| miR1135 | P51WP25427^R^ | miR1135 | UGCGACAAGUAAUUCCGGACGGAG | 24 |  |  |  | (07)-miR1135 [3] |
|  |  |  |  |  |  |  |  |  |
| miR1137 | TF6215B31467^R^ | miR1137 | UAGUACAAAGUUGAGACAGUU | 21 |  |  |  | (07)-miR1137 [3] |
|  |  |  |  |  |  |  |  |  |
| miR1318/ miR1432 | GPB339 | miR1318a | UCAGGAGAGAUGACACCGACA | 21 | 3763 | miR1318 [1];  miR1432 [2] |  | (09)-miR1432 [2] |
|  | GPA513 | miR1318b | AUCAGGAGAGAUGACACCGG | 20 | 2600 | miR1318 [3] |  |  |
|  |  |  |  |  |  |  |  |  |
| miR1426 | GPB17479^R^ | miR1426 | AGAAUCUUGAUGAUGCUUCAU | 21 | 116 | miR1426 [3] |  |  |
|  |  |  |  |  |  |  |  |  |
| miR1436 | P45NP8483^R^ | miR1436a | AUAUUAUGGGACGGAGGGAGU | 21 | 97 | miR1436 [1] |  |  |
|  | P45NP24425^R^ | miR1436b | UACAUUAUGGGACGGAGGGAG | 21 | 42 | miR1436 [2] |  |  |
|  | GPB40621^R^ | miR1436c | AUAUUUUGGAACGGAGGGAGU | 21 | 23 | miR1436 [3] |  |  |
|  |  |  |  |  |  |  |  |  |
| miR2002 | GPB12064^P^ | miR2002 | UGAGAUGAGAUUACCCCAUAC | 21 | 22 |  |  | (09)-miR2002 |
|  |  |  |  |  |  |  |  |  |
| miR2003 | GPB2402 | miR2003 | CGGUAGGGCUGUAUGAUGGCGA | 22 | 178 |  |  | (09)-miR2003 [1] |
|  |  |  |  |  |  |  |  |  |
| miR2004 | P45NP3963^NH^ | miR2004 | UUUGUUUUUAUGUUAUUUUGUGAAG | 25 | 95 |  |  | (09)- miR2004 [1] |
|  |  |  |  |  |  |  |  |  |
| miR2005 | TF6215B52^NH^ | miR2005a | GCGGGGAUAGCUCAGUUGG | 19 | 30007 |  |  | (09)-miR2005 [3] |
|  | GPA638^R^ | miR2005b | GGGGAUAUAGCUCAGUUGG | 19 | 2391 |  |  | (09)-miR2005 [2] |
|  |  |  |  |  |  |  |  |  |
| miR2006 | GPB969^NH^ | miR2006 | UACCACGACUGUCAUUAAGCA | 21 | 409 |  |  | (09)- miR2006 |
|  |  |  |  |  |  |  |  |  |
| miR2007 | TF6215B9441^NH^ | miR2007 | CAAGAUAUUGGGUAUUUUGAA | 21 | 145 |  |  | (09)- miR2007 [3] |
|  |  |  |  |  |  |  |  |  |
| miR2008 | GPA1514^R^ | miR2008a | GACCGCGUGGCCUAAUGGA | 19 | 385 |  |  | (09)- miR2008 [3] |
|  | GPA2491^R^ | miR2008b | GACUCCGUGGCCCAAUGGA | 19 | 266 |  |  | (09)- miR2008 |
|  |  |  |  |  |  |  |  |  |
| miR2009 | GPB159 | miR2009d | UGAGAAGGCAGAUCAUAAUAGC | 22 | 10206 |  |  | (09)-miR2009a [1] |
|  | GPB2271 | miR2009c | UCAGAUGAGAAGGCAGAUCAUA | 22 | 370 |  |  | (09)-miR2009c |
|  | GPB1100 | miR2009a | UGAGAAGGUAGAUCAUAAUAGC | 22 | 334 |  |  | (09)-miR2009a |
|  | GPA12904 | miR2009e | UUCAGAUGAGAAGGCAGAUCA | 21 | 84 |  |  | (09)-miR2009b [3] |
|  | GPB13969 | miR2009b | UUAGAUGAGAAGGCAGAUCAUA | 22 | 20 |  |  | (09)-miR2009b |
|  |  |  |  |  |  |  |  |  |
| miR2011 | GPB3564 | miR2011 | UGCAGUGGCAUAUGCAACUCU | 21 | 194 |  |  | (09)-miR2011 |
|  |  |  |  |  |  |  |  |  |
| miR2012 | P45NP2802^R^ | miR2012a | UUGGACGUGGAGGUGCAGCUG | 21 | 1252 |  |  | (09)-miR2012 [3] |
|  | GPB14783^R^ | miR2012b | UGGACGAGGAUGUGCAACUGC | 21 | 19 |  |  | (09)-miR2012 [2] |
|  |  |  |  |  |  |  |  |  |
| miR2016 | P45NP4027^NH^ | miR2016 | UGACCCUGAGGCACUCAUACCG | 22 | 156 |  |  | (09)-miR2016 |
|  |  |  |  |  |  |  |  |  |
| miR2018 | P45NP5575^R^ | miR2018a | GCCCCUGUAGCUCAGUUGGU | 20 | 175 |  |  | (09)-miR2018 [2] |
|  | P45NP3381^R^ | miR2018b | GCCCGUCUAGCUCAGUUGGU | 20 | 173 |  |  | (09)-miR2018 |
|  |  |  |  |  |  |  |  |  |
| miR2020 | GPB7767^NH^ | miR2020a | AUAGCAUCAUCCAUCCUACCC | 21 | 46 |  |  | (09)-miR2020 [1] |
|  | GPB9373^NH^ | miR2020b | AUAGCAUCAUCCAUCCUACCA | 21 | 35 |  |  | (09)-miR2020 |
|  |  |  |  |  |  |  |  |  |
| miR2032 | GPB7405 | miR2032 | UGUAGAUACUCCCUAAGGCUU | 21 | 60 |  |  | (09)-miR2032 |
|  |  |  |  |  |  |  |  |  |
| miR2033 | GPA19347^R^ | miR2033a | UAUUUCGGUACAGAGGGAGUA | 21 | 36 |  |  | (09)-miR2033a [2] |
|  | GPA21162^NH^ | miR2033b | UAUUUCGGUACAGAGGUAGUA | 21 | 21 |  |  | (09)-miR2033a [3] |
|  |  |  |  |  |  |  |  |  |
| miR2502 | P51WP36799^NH^ | miR2502 | CGUCAAUUAAUUCGGGUUGGA | 21 | 25 |  | miR2502 [3] |  |
|  |  |  |  |  |  |  |  |  |
| miR2509 | P45NP2560 | miR2509 | GAAUGACGCCGGGUCCGAAAG | 21 | 261 |  | miR2509 [1] |  |

^a^A superscript next to a read name indicates that this sequence was not classified as a potential miRNA in our dataset because if failed our filtering procedure (R: repetitive sequence or rRNA; NH: no valid hairpin; P: abundance-profile not classified as miRNA-like). Sequences that are likely to be sequencing errors as well as those with total abundance less than 12 (see text) are not listed.

^b^Numbers in square brackets indicate the number of mismatches between the reads and existing sequences.

^c^For Brachypodium, (U)- refers to a sequence described in [38], all others are those from [33].

^d^(07) and (09) indicates that the miRNA was only described in [14] or [33], respectively.
