# Supplementary material for: Discovery of barley miRNAs through deep sequencing of short reads
Source: BMC Genomics. 2011 Feb 25;12:129. doi: 10.1186/1471-2164-12-129 (PMC3060140; doi:10.1186/1471-2164-12-129)
Supplement: Additional file 3 — Secondary structure of putative hairpin orthologs for the barley miRNA candidates listed in Table 6. Only the most compact hairpin in the species most closely related to barley is shown and annotated with species, chromosome/accession ID, orientation and location of the match (Notation: osa and bdi-rice and Brachypodium genome, respectively; Ta and Hv BAC-wheat and barley BAC sequences, respectively; Hv TC-barley tentative contigs). The location of the approximate match of the barley miRNA candidate on the hairpin is indicated by a solid bar. [file 1471-2164-12-129-S3.PDF]

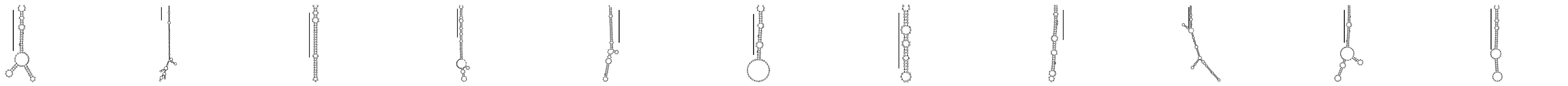

GPB235	GPB125	GPB1131	P51WP1692	GPB8582	GPB86	GPA5819	GPB2903	GPA3884	P45NP9164	P45NP15764
bdi	Hv TC	Hv TC	bdi	Hv TC	osa	osa	bdi	bdi	bdi	osa
super2 +/+	TC192947 +/+	TC169777 +/-	super2 +/-	AV836089 +/-	chr8 +/-	chr6 +/-	super3 +/+	super3 +/+	super2 +/-	chr8 +/+
21931634-21931655	201-222	993-1013	21726084-21726104	412-432	4113074-4113093	17457741-17457762	16838844-16838861	9491757-9491775	11167594-11167614	19017792-19017814

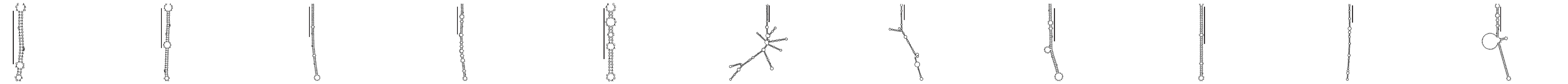

GPB4154	TF6215B7984	TF6215B74145	TF6215B6553	P51WP57134	P51WP4847	P51WP10727	P45NP51144	P45NP40836	P45NP31207	P45NP21816
bdi	bdi	Ta BAC	osa	bdi	osa	bdi	bdi	Hv TC	osa	osa
super6 +/-	super7 +/-	gi|21779916 +/+	chr12 +/+	super1 +/+	chr11 +/-	super2 +/+	super1 +/+	TC163338 +/+	chr1 +/-	chr4 +/-
10281542-10281562	17417805-17417826	59711-59731	12671865-12671885	15905534-15905553	7874091-7874111	994310-994329	22735066-22735086	1284-1304	43207588-43207608	259944-259964

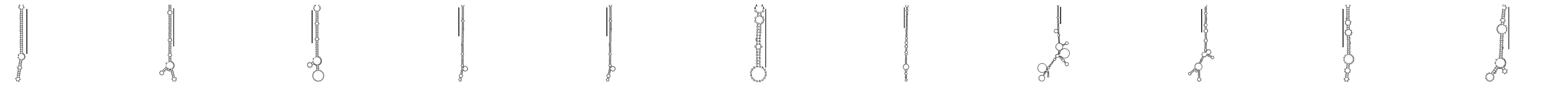

P45NP16270	GPB49459	GPB41819	GPB2977	GPB5902	GPB16764	P51WP18432	GPB1614	GPB25359	GPB17570	GPB26526
bdi	Ta BAC	Ta BAC	bdi	bdi	bdi	Hv TC	bdi	bdi	bdi	Hv TC
super7 +/-	gi|139096360 +/+	gi|154937437 +/+	super0 +/+	super0 +/+	super0 +/+	TC182201 +/+	super4 +/+	super5 +/+	super1 +/-	TC179520 +/-
11530363-11530386	61565-61588	76666-76686	14989573-14989593	14989573-14989593	4563002-4563023	742-762	9800455-9800474	831891-831911	16482659-16482680	207-227

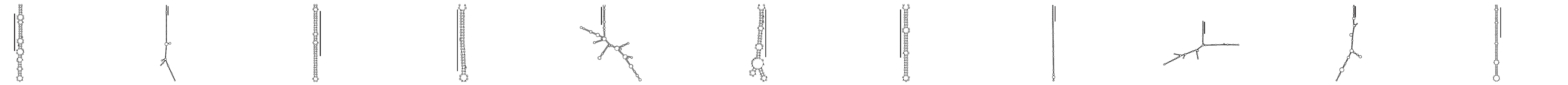

GPB320	P45NP187203	GPB373	P45NP20958	P45NP15484	GPB9781	GPB37150	GPA47901	GPA42571	GPA18850	GPA14470
osa	osa	bdi	bdi	osa	osa	bdi	osa	osa	osa	bdi
chr3 +/+	chr5 +/-	super0 +/-	super3 +/+	chr6 +/+	chr10 +/-	super12 +/-	chr7 +/-	chr11 +/+	chr1 +/-	super4 +/+
14093451-14093471	21845314-21845332	18438564-18438583	20782702-20782725	28180420-28180441	2321007-2321027	5482078-5482098	15898-15919	1285655-1285677	41009540-41009559	6021072-6021092
